# Endothelial activation and dysfunction in severe fever with thrombocytopenia syndrome

**DOI:** 10.1371/journal.pntd.0005746

**Published:** 2017-08-14

**Authors:** Xiao-Kun Li, Zhen-Dong Yang, Juan Du, Bo Xing, Ning Cui, Pan-He Zhang, Hao Li, Xiao-Ai Zhang, Qing-Bin Lu, Wei Liu

**Affiliations:** 1 State Key Laboratory of Pathogen and Biosecurity, Beijing Institute of Microbiology and Epidemiology, Beijing, P. R. China; 2 The 154 Hospital, People’s Liberation Army, Xinyang, P. R. China; 3 Department of Laboratorial Science and Technology, School of Public Health, Peking University, Beijing, P. R. China; 4 Beijing Key Laboratory of Toxicological Research and Risk Assessment for Food Safety, Beijing, P. R. China; University of Rhode Island, UNITED STATES

## Abstract

**Background:**

Pathogenesis of severe fever with thrombocytopenia syndrome (SFTS) has not been well described yet. Recent studies indicate that SFTSV could replicate in endothelial cells. Here we performed a case-control study to determine whether endothelial activation/dysfunction occurred in SFTSV infection and to identify the biomarkers reflecting endothelial dysfunction.

**Methodology/Principal findings:**

In a case-control study of 134 SFTS patients and 68 healthy controls, serum levels of plasminogen activator inhibitor 1, tissue plasminogen activator, P-selectin, platelet endothelial cell adhesion molecular, CD40 ligand, E-selectin, vascular endothelial growth factor A, serum amyloid antigen 1 (SAA-1) and vascular cell adhesion molecular 1 were significantly enhanced in the patients than the controls (all P<0.05), indicating the occurrence of endothelial activation/dysfunction in SFTS. The intercellular adhesion molecular 1 (ICAM-1) and SAA-1 at the convalescent phase were also significantly associated with severe patients, after adjusting for the potential confounders. The odds ratio was estimated to be 3.364 (95% CI 1.074–10.534) for ICAM-1, and 1.881 (95% CI 1.166–3.034) for SAA-1, respectively. Cutoff value of 1.1×10^7^ pg/mL SAA-1 or 1.2×10^6^ pg/mL ICAM-1 were found to have moderate power of predicting fatal cases.

**Conclusions:**

The endothelial dysfunction may be one of the pathogenic mechanism of SFTS. The serum levels of ICAM-1 and SAA-1 might be used to predict adverse outcome.

## Author summary

Severe fever with thrombocytopenia syndrome (SFTS) is a tick-borne viral disease and first reported in the rural areas of China. Pathogenesis of the disease has not been well described yet. Recent studies indicated that SFTSV replicated in endothelial cells. So, we performed a case-control study to explore whether endothelial activation/dysfunction occurred in SFTSV infection and to identify biomarkers reflecting endothelial dysfunction. We found that the occurrence of endothelial activation/dysfunction in severe fever with thrombocytopenia syndrome and the serum levels of ICAM-1 and SAA-1 might be used to predict adverse outcome.

## Introduction

Severe fever with thrombocytopenia syndrome (SFTS) is a tick-borne viral disease that is caused by a novel bunyavirus, SFTSV, which was first reported in the rural areas of China [1]. A genetically similar virus, heartland virus was also demonstrated to cause mortality in USA [2,3]. The disease is clinically characterized by fever, thrombocytopenia and leucopenia, with the mortality rate varying between 10% and 30% in different studies. The severe cases could present with hemorrhagic, neurologic, multiple organs dysfunction, and even developing fatal outcome [1,4]. Pathogenesis of the disease has not been well described yet. Recent studies indicate that SFTSV could replicate in endothelial cells [4] and it’s hypothesized that SFTSV-infected endothelial cells may directly contribute to viremia, vascular permeability. On the other hand, it’s suggested that SFTSV might also mediate endothelial cell activation via an indirect route, considering the significant elevation of circulating TNF-α in SFTS cases [5,6], a strong activator of vascular endothelium. Endothelial dysfunction usually includes several proinflammatory and procoagulant changes as well as endothelial activation [7]. Here in order to determine whether endothelial activation/dysfunction occurred in SFTSV infection and to identify biomarkers reflecting endothelial dysfunction that can be used to predict disease outcome in SFTSV infection, we performed a case-control study in PLA 154 hospital in Xinyang City, Henan Province, China. The serum levels of endothelial function makers were evaluated and compared regarding their clinical progression and disease severity.

## Methods

### Subjects

The SFTS patients who were treated in PLA154 hospital during 2015–2016 were included in this study. SFTSV infection was confirmed by real-time reverse transcription-polymerase chain reaction (RT-PCR) tests or serological tests as guided by the Ministry of Health, China [8]. The serum samples that were collected on admission were used as acute phase samples, and all were within seven days after the onset of disease. The second samples were collected when the patients had their clinical manifestations resolved and the main laboratory abnormalities (decreased white blood cell and platelet counts, increased transaminase) restored to normal. For the fatal patients, the second samples were collected at the time of disease deterioration. Severe patients were defined by the presence of hemorrhagic manifestations (melena, hematemesis, hemoptysis, ophthalmorrhagia and gingival bleeding), any one or more organ failure or encephalitis development [9].Healthy blood donors with comparable age and gender who were determined to be SFTSV negative by both real-time RT-PCR test and serological test were enrolled as controls from the department of physical examination in the same hospital. After the participants were enrolled, their underlying disease was further checked and those with hypertension or cardiovascular diseases were excluded from the study, due to the known endothelial activation/dysfunction that occurred in these diseases.

### Ethics statement

The study was approved by the ethics committee of PLA 154 hospital. All participants gave written informed consent.

### Detection of cytokines and adhesion molecules reflecting endothelial dysfunction

Serum levels of vascular endothelial growth factor A (VEGF-A), P-selectin, E-selectin, platelet endothelial cell adhesion molecular (PECAM-1), CD40 ligand (CD40L), tissue plasminogen activator (tPA), plasminogen activator inhibitor 1 (PAI-1), serum amyloid antigen 1 (SAA-1), vascular cell adhesion molecular 1 (VCAM-1) and intercellular adhesion molecular 1 (ICAM-1) were determined by the ProcartaPlex multiplex immunoassays panels (Affymetrix, USA) according to the manufacturer instructions. Each serum sample was tested in duplicates and the concentration was further determined according to the dilution curve of standard materials.

### Statistical analysis

Serum levels of the above-mentioned molecules reflecting endothelial dysfunction were compared between groups after performing base 10 logarithmic transformations. For variables that were not normally distributed, comparisons were made with the Mann-Whitney U test. The logistic regression model was performed to explore the correlations introduced by estimating the odds ratios (ORs) and 95% confidence intervals (CIs) for levels of biomarkers predicting severe and fatal SFTS patients. Receiver operator characteristic (ROC) curves were constructed and the area under the ROC curve (AUC) was calculated as a measure of discriminative ability. *P*-value of <0.05 was considered statistically significant. All analyses were performed using SAS 9.1.3 (SAS Institute Inc., Cary, NC, USA).

## Results

### The subjects

Among all 219 patients with clinical suspicion of SFTS, 134 patients were confirmed to be infected with SFTSV and included into the study, their mean age was 61 years (Standard deviation, 11 years) and 45 (33.6%) were male. The demographic characteristics were comparable with those of the 68 controls (age 58±17 years, male gender 42.7%, P = 0.132 and 0.206, respectively). Fifty-seven (42.5%) patients had severe outcome and 12 (9.0%) patients died. The median duration between the disease onset and the day in which acute serum samples were collected was 6 days (interquartile range 5–7 days). The severe and mild patients had their convalescence samples collected at 16 (interquartile range 14–18) days and 14 (interquartile range 12–16) after disease onset, respectively. For the fatal cases, the median duration between the disease onset and the day in which the second sample was collected was 12 days (interquartile range 10–14 days). The clinical information of the SFTS patients were listed in Table 1.

**Table 1:** The characteristics and clinical manifestations of SFTS patients.

Characteristic	SFTS patients (n = 134)
**Demographic characteristics**	
Male gender/ No. (%)	45 (33.6)
Age, y, mean±SD	61±11
Days delay, median (IQR)	6 (5–7)
Hospital duration, median (IQR)	16 (14–17)
**Clinical manifestations**	
Fever >38°C	134 (100)
Gastrointestinal syndromes	121 (90.3)
Nausea	118 (88.1)
Vomiting	49 (36.6)
Diarrhea	38 (28.4)
Neurological symptoms	37 (27.6)
Dysphoria	15 (11.2)
Convulsion	27 (20.2)
Clouding of consciousness	22 (16.4)
Lethargy	5 (3.7)
Coma	7 (5.3)
Hemorrhagic manifestation	34 (25.4)
Melena	11 (8.2)
Hematemesis	1 (0.8)
Hemoptysis,	7 (5.2)
Gingival bleeding	21 (15.7)
Malaise	134 (100)
Lymphadenectasis	57 (42.5)
Cough	95 (70.9)
Sputum production	74 (55.2)
Dizzy	24 (17.9)
Headache	18 (13.4)
Dyspnea	4 (3.0)
Severe	57 (42.5)
Fatal	12 (9.0)
**Laboratory features on admission**	
White blood cell count < 4×10^9^/L	115 (85.8)
Platelet count < 100 ×10^9^/L	110 (82.1)
Neutrophils > 70%	68 (50.8)
Lymphocytes < 20%	57 (42.5)
Hemoglobin < 110 g/L	22 (16.4)
Aspartic transaminase> 40 U/L	114 (85.1)
Alanine aminotransferase > 40 U/L	72 (53.7)
Total protein <60 g/L	15 (11.2)
Albumin < 35 g/L	11 (8.2)
Alkaline phosphatase > 150 U/L	3 (2.2)
Gamma-glutamyl transpeptidase >50 U/L	23 (17.2)
Lactate dehydrogenase > 245 U/L	118 (88.1)
Creatine kinase >200 U/L	85 (63.4)
Urea nitrogen >7.14 mmol/L	50 (37.3)
Total bilirubin >17.1 μmol/L	4 (3.0)
Creatinine > 97 μmol/L	22 (16.4)
Amylase > 115 U/L	32 (23.9)

### Comparison between case and control groups

**Fig 1:**
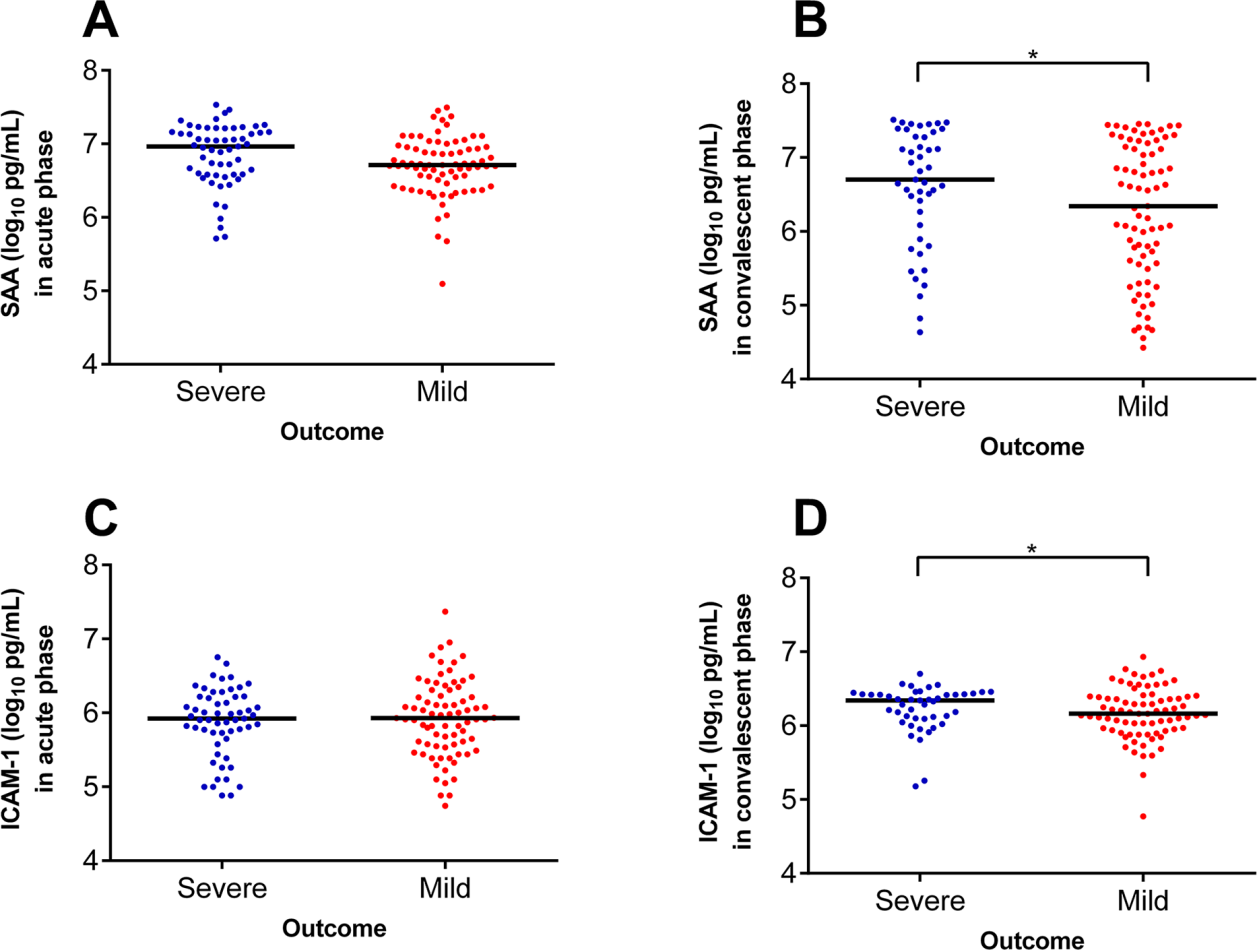
The distribution of serum amyloid A proteins 1 (SAA-1) and intercellular adhesion molecular 1 (ICAM-1) between severe and mild patients at acute phase. The comparison was performed by adjusting the effect from age, sex, the interval from disease onset to admission and underlying diseases. A, SAA-1 in acute phase; B, SAA-1 in convalescent phase; C, ICAM-1 in acute phase; D, ICAM-1 in convalescent phase.

Nine of the 10 parameters that were evaluated at acute phase of SFTSV infection, i.e., PAI-1, tPA, P-selectin, PECAM-1, CD40L, E-selectin, VEGFA, SAA-1 and VCAM-1 were increased to significantly higher levels in the cases than in the controls. Only ICAM-1 was comparable between two groups (Table 2). At convalescence, all the 10 parameters were found to be significantly different between cases and controls. When the evaluations were compared between severe and mild patients, only the ICAM-1 and SAA-1 at the convalescent phase showed significantly higher levels in severe patients than in the mild patients after multivariate analysis (P = 0.004 and 0.010 respectively, Table 3 and Fig 1). The ORs of severe outcome was 3.364 (95% CI 1.074–10.534) for ICAM-1 (log_10_-transformed, pg/mL), and 1.881 (95% CI 1.166–3.034) for SAA-1 (log_10_-transformed, pg/mL) (Table 4). All the other evaluations were comparable between two groups, by applying multivariate analysis to adjust the effect from the age, sex the interval from disease onset to admission and underlying diseases (Table 3).

**Table 2:** The levels of the cytokines and adhesion molecules reflecting endothelial dysfunction in cases and controls.

Biomarkers (pg/mL)	Case group		Control group (n = 68)	P value		
	Acute sampling (n = 134)	Second sampling (n = 122)		Acute vs. Second	Acute vs. Control	Second vs. Control
VEGFA	2.74 (2.52, 3.01)	2.92 (2.55, 3.11)	1.14 (0.21, 2.65)	0.033	<0.001	<0.001
ICAM-1	5.92 (5.57, 6.22)	6.24 (6.03, 6.44)	6.06 (5.84, 6.23)	<0.001	0.073	<0.001
CD40L	0.93 (0.13, 1.72)	1.69 (0.75, 2.07)	-0.22 (-0.27, 1.53)	<0.001	<0.001	<0.001
PAI-1	4.61 (4.42, 4.77)	4.37 (4.10, 4.66)	3.38 (1.01, 4.07)	<0.001	<0.001	<0.001
tPA	3.76 (3.26, 4.04)	3.19 (2.78, 3.76)	2.22 (1.97, 3.12)	<0.001	<0.001	<0.001
VCAM-1	5.94 (5.83, 6.1)	5.87 (5.78, 5.94)	5.82 (5.68, 5.89)	<0.001	<0.001	0.013
P-selectin	4.12 (3.87, 4.31)	4.19 (3.93, 4.4)	3.9 (3.43, 4.36)	0.068	0.011	<0.001
PECAM-1	4.53 (4.3, 4.68)	4.46 (4.17, 4.71)	4.07 (3.34, 4.42)	0.081	<0.001	<0.001
E-selectin	3.99 (3.7, 4.19)	3.99 (3.63, 4.25)	3.36 (3.01, 3.95)	0.480	<0.001	<0.001
SAA-1	6.74 (6.55, 7.07)	6.66 (5.8, 7.28)	5.05 (4.71, 5.32)	0.064	<0.001	<0.001

Data are given as log-transformed median (interquartile range); The comparison was made by applying Mann-Whitney U test.

**Table 3:** The levels of the cytokines and adhesion molecules reflecting endothelial dysfunction in the severe patients and mild patients.

Biomarkers (pg/mL)	Acute phase of SFTS			Convalescent phase of SFTS		
	Severe (n = 57)	Mild (n = 77)	P	Severe (n = 45)	Mild (n = 77)	P
tPA	3.71 (3.31, 3.99)	3.79 (3.26, 4.07)	0.697	3.08 (2.78, 3.65)	3.42 (2.78, 3.77)	0.160
PAI-1	4.61 (4.43, 4.77)	4.61 (4.41, 4.76)	0.822	4.5 (4.06, 4.66)	4.33 (4.11, 4.67)	0.737
P-selectin	4.12 (3.86, 4.28)	4.12 (3.91, 4.33)	0.381	4.2 (3.9, 4.42)	4.19 (3.97, 4.39)	0.754
PECAM-1	4.43 (4.27, 4.65)	4.56 (4.37, 4.7)	0.090	4.45 (4.17, 4.85)	4.47 (4.21, 4.67)	0.518
CD40L	0.42 (0.13, 1.59)	1.21 (0.13, 1.72)	0.051	1.43 (0.31, 1.96)	1.8 (1.23, 2.18)	0.022
E-selectin	3.98 (3.63, 4.19)	4 (3.73, 4.18)	0.729	3.98 (3.58, 4.26)	4.02 (3.73, 4.23)	0.792
VEGFA	2.71 (2.51, 2.92)	2.75 (2.58, 3.02)	0.283	2.89 (2.22, 3.11)	2.93 (2.69, 3.12)	0.357
SAA-1	6.96 (6.58, 7.16)	6.71 (6.46, 6.95)	0.098	7.01 (6.42, 7.34)	6.34 (5.57, 7.14)	0.004
ICAM-1	5.92 (5.73, 6.22)	5.93 (5.57, 6.23)	0.724	6.36 (6.13, 6.46)	6.16 (5.95, 6.39)	0.010
VCAM-1	5.92 (5.82, 6.05)	5.95 (5.85, 6.13)	0.230	5.87 (5.76, 5.95)	5.87 (5.79, 5.94)	0.632

Data are given as log-transformed median (interquartile range); The comparison was made by applying Mann-Whitney U test.

**Table 4:** The association between the cytokines and adhesion molecules reflecting endothelial dysfunction and severe outcome.

Biomarkers (pg/mL)	Severe	Mild	Crude			Adjusted*		
			OR	95%CI	P	OR	95%CI	P
**Acute phase**								
SAA-1	6.96 (6.58, 7.16)	6.71 (6.46, 6.95)	2.584	1.061–6.339	0.037	2.168	0.868–5.419	0.098
**Convalescent phase**								
CD40L	1.43 (0.31, 1.96)	1.80 (1.23, 2.18)	0.648	0.433–0.970	0.035	0.666	0.438–1.011	0.056
ICAM-1	6.36 (6.13, 6.46)	6.16 (5.95, 6.39)	3.386	1.123–10.209	0.030	3.364	1.074–10.534	0.037
SAA-1	7.01 (6.42, 7.34)	6.34 (5.57, 7.14)	1.861	1.211–2.859	0.005	1.881	1.166–3.034	0.010

Note: The ORs (95% CI) were calculated using the log_10_-transformed data of the biomarkers.

*adjusted for the variables of age, sex the interval from disease onset to admission and underlying diseases.

The levels of PAI-1, tPA and VCAM-1 were significantly decreased in convalescence compared with the acute samples, however, remained significantly different from those of the controls (Table 2). In contrast, the level of CD40L and VEGFA were further increased, with the convalescence level were significantly higher, even than the acute phase (Table 2).

**Fig 2:**
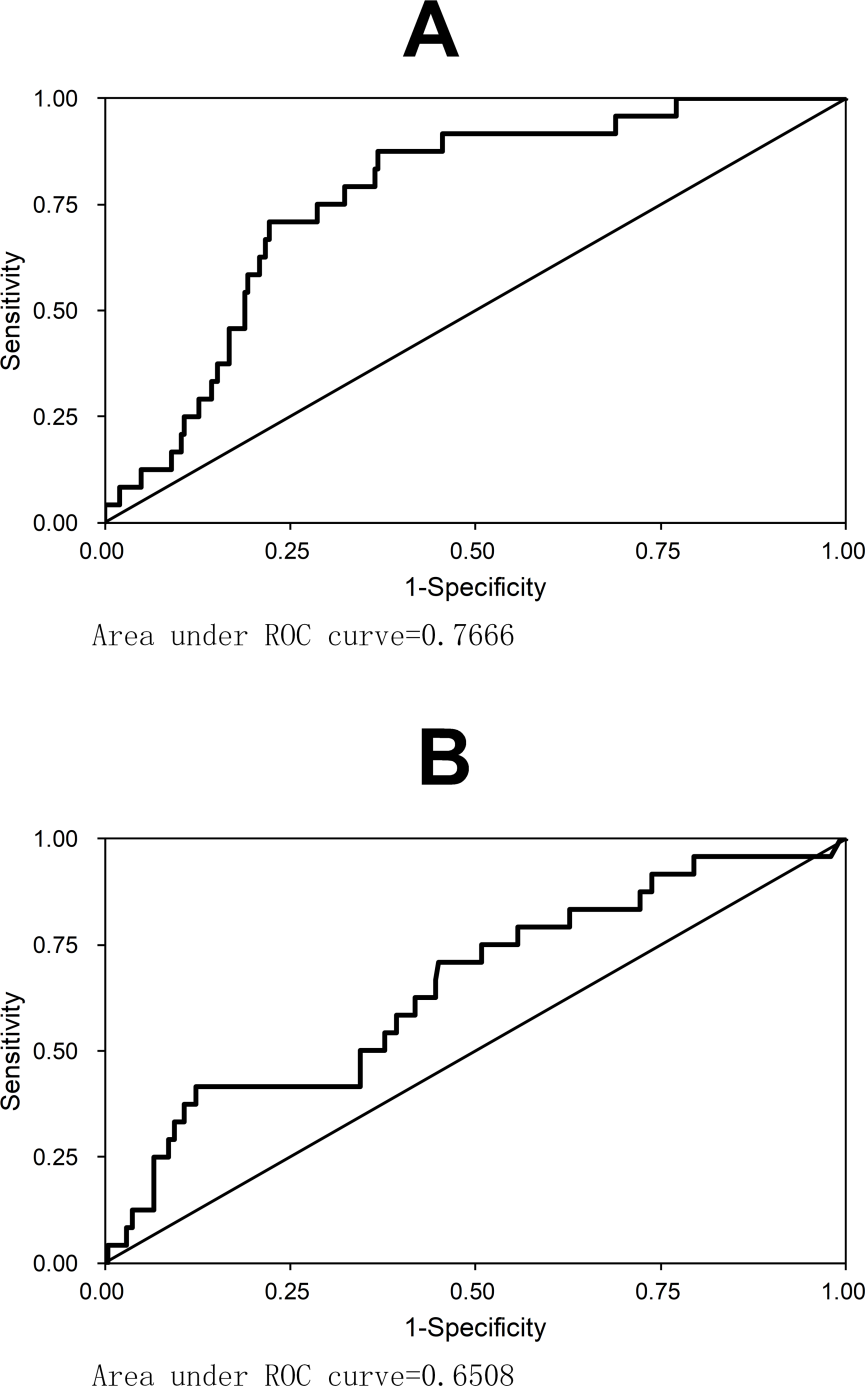
The ROC curves of SAA-1 (A) and ICAM-1 (B) for predicting mortality in SFTS patients.

It’s interesting that the ICAM-1, although comparable between cases and controls, turned to be increased to higher than the controls (Table 1), and even higher in severe patients than in mild patients (Fig 1). When the fatal cases were analyzed separately, the levels of ICAM-1 and SAA-1 in second samples of fatal cases were found to be even more significantly increased compared to the non-fatal patients in the convalescent phase (Table 5). ROC analysis demonstrated the good diagnostic accuracy for SAA-1 (AUC = 0.767) and ICAM-1 (AUC = 0.651) to the prediction of mortality (Fig 2). The SAA-1 had 75.0% sensitivity and 71.3% specificity for prediction of mortality at a cutoff of 1.1×10^7^ pg/mL; and the ICAM-1 had 75.0% sensitivity and 49.2% specificity for prediction of mortality at a cutoff of 1.2×10^6^ pg/mL. These results indicated these two parameters could be used as consistent markers as to differentiate the cases from controls, and predicting the severe patients and even fatal patients.

**Table 5:** The association between the cytokines and adhesion molecules reflecting endothelial dysfunction and fatal outcome of SFTS patients.

Biomarkers (pg/mL)	Fatal	Non-Fatal	Crude			Adjusted*		
			OR	95%CI	P	OR	95%CI	P
**Acute phase**								
CD40L	-.027 (-0.27, 0.48)	1.05 (0.13, 1.74)	0.223	0.071–0.697	0.010	0.218	0.067–0.713	0.012
PECAM-1	4.18 (3.13, 4.26)	4.56 (4.36, 4.69)	0.001	<0.001–0.026	<0.001	<0.001	<0.001–0.006	<0.001
E-selectin	3.60 (3.33, 3.80)	4.01 (3.73, 4.21)	0.041	0.007–0.240	<0.001	0.032	0.005–0.239	0.001
VEGFA	2.41 (2.07, 2.55)	2.78 (2.58, 3.02)	0.032	0.005–0.191	<0.001	0.017	0.002–0.138	<0.001
SAA-1	7.13 (6.95, 7.22)	6.72 (6.54, 7.05)	13.277	1.754–100.472	0.012	11.409	1.493–87.183	0.019
**Fatal cases at deterioration vs. non-fatal cases in convalescent phase**								
CD40L	-0.27 (-0.27, 1.32)	1.74 (1.09, 2.12)	0.185	0.075–0.456	<0.001	0.181	0.068–0.483	0.001
ICAM-1	6.61 (6.34, 6.69)	6.21 (6.02, 6.42)	129.446	7.402–2263.522	0.001	122.669	6.609–2276.791	0.001
VEGFA	1.98 (1.64, 2.56)	2.93 (2.65, 3.12)	0.117	0.038–0.357	<0.001	0.120	0.040–0.365	<0.001
SAA-1	7.30 (7.22, 7.37)	6.60 (5.73. 7.22)	8.306	1.566–44.045	0.013	8.166	1.356–49.189	0.022

Note: The ORs (95% CI) were calculated using the log_10_-transformed data of the biomarkers.

*adjusted for the variables of age, sex the interval from disease onset to admission and underlying diseases.

## Discussion

In this case control study, we identified that elevated levels of adhesion molecule were associated with SFTS disease. All these elevated plasma measurements could be considered as the marker of endothelial activation/dysfunction; therefore, we suggest that the endothelial activation/dysfunction might act as one of the pathogenesis of SFTSV infection. We also found that serum levels of ICAM-1 and SAA-1 might be an indicator of adverse prognosis in SFTS, independent of other impact factors. Using a discrimination analysis, we are able to infer a cutoff value that might predict the occurrence of fatal SFTS.

Like several previous reports, the serum level of ICAM-1 alone, but not E-selectin or VCAM-1 was found to be an independent predictor of severity or mortality in the cases [10,11]. ICAM-1 is the member of immunoglobulin superfamily, which is expressed by leucocytes and the endothelial cells of vessels. ICAM-1 is present at very low levels in healthy humans. Their concentration might be increased markedly in various inflammatory conditions [12,13]. Levels of ICAM-1 were even higher in severe and fatal cases in late disease, which may related to the adverse outcome.

Consistent with this alteration is the increased SAA-1 levels in patient, also seen in those severe patients. SAA-1 has also been proven for its potent impact on the dysfunction of endothelial, through the suppression of endothelial nitric oxide synthase function and endothelial-derived nitric oxide bioviability [14]. The higher expression of ICAM-1 has been linked to the systematic injuries of vascular endothelial cells in the LPS stimulated disseminated intravascular coagulation (DIC) mice model [15], and SAA-1 was found to disturb the balance of endothelial tissue factor pathway, which acts as a component of initiation of coagulation cascade [16]. Similarly, these two indicators, through mediating the endothelial damage and activation, contribute to hemostatic failure by stimulating platelet aggregation and degranulation, resulting in consequent activation of the intrinsic coagulation cascade [7]. This is consistent with the late development of DIC in most of the severe and fatal SFTS cases [17].

VEGFA is known to be the most specific mitogenic factor of endothelial cells. It is originally found as a vascular permeability factor that causes vascular leakage. In the Dengue virus study, an increased level of VEGFA associated with a decrease in its soluble receptor, VEGFR2 were observed in patients with Dengue hemorrhagic fever [18], and the significant elevation of VEGFA in the acute phase may directly indicate the generalized capillary damage and vascular leakage [19]. In the current SFTS patients, a prolonged high level of VEGFA in the recovery period may indicate the sustained vascular angiogenesis and repairmen, similar with hantavirus infection, also from the Bunyaviridae [20].

The role of vascular endothelium in the pathogenesis of vascular disease has been better known in the last 30 years, and also been increasingly related to the infectious disease, especially bacterial sepsis and viral hemorrhagic fever [21]. As displayed in previous findings, endothelial activation/dysfunction has an important role in the pathogenesis of known members of viral hemorrhagic fever virus, such as CCHF, Ebola virus. In case of vascular dysregulation and hyperpermeability, patients might exhibit thrombocytopenia, leukopenia, proteinuria, and fluid distribution problems. Consistent with previous findings, all these symptoms have occurred in SFTSV infection and clinically related to more severe disease.

Several investigations have performed in the SFTS patients or animal models to determine whether circulating cytokines and chemokines have their diagnostic or prognostic value, but only yielding conflicting results [5,22,23]. These cytokines and chemokines displayed rapid alteration and recovery in the viral hemorrhagic fever conditions, especially in severe cases, therefore their levels are remarkably influenced by the sampling time. In contrast, the measurement of adhesion molecules, like SAA-1 and ICAM-1 would have a better reproducibility in the correlation with the disease, due to their persistent alteration after disease [14].

Our study has limitation that we assessed endothelial dysfunction solely by means of serum biomarkers and do not have other measures of vascular physiology, which needs further investigation. Despite of this limitation, through a relatively large study population, we found that elevated plasma levels of biomarkers reflecting endothelial activation/dysfunction were observed in SFTSV infection, and independently associated with severe disease outcome after SFTSV infection. As the significant differences were only seen at later phase of infection, the predictive use of these biomarkers was restricted. More studies were needed to further demonstrate the dynamic profile of the biomarkers.

The occurrence of endothelial dysregulation, which is a common feature of viral hemorrhagic fever, also developed in SFTS. Thus, similar with other known viral hemorrhagic fever, endothelial dysfunction might be one of the pathogenic mechanism of SFTS, and the therapies that control endothelial dysfunction might be applied in the treatment of SFTS.
